# Trabeculectomy: Does It Have a Future?

**DOI:** 10.7759/cureus.27834

**Published:** 2022-08-09

**Authors:** Aparna Rao, Rakhi D Cruz

**Affiliations:** 1 Glaucoma, L V Prasad Eye Institute, Mithu Tulsi Chanrai Campus, Bhubaneswar, IND

**Keywords:** non-penetrating glaucoma surgery, phacotrabeculectomy, minimally invasive glaucoma surgeries, glaucoma surgery, trabeculectomy

## Abstract

The trabeculectomy (TRAB) procedure has undergone various modifications to increase the long-term surgical success and safety profiles. The main issues with TRAB include short and long-term complications, that are more common with the concomitant use of anti-fibrotic agents. While many surgeons have predicted the demise of trabeculectomy amidst newer non-penetrating glaucoma surgeries, it is still the gold standard procedure for patients with an advanced or rapidly progressing disease and for those patients who need very low intraocular pressures. This review article is unique in summarizing the evolution of trabeculectomy and its efficacy compared to neoteric shunt procedures while trying to predict if trabeculectomy has a future in the modern surgical world. We have compared the outcomes and complications of trabeculectomy to all the surgical procedures available to date and have tried to evolve an algorithm to help surgeons to decide on their preferred technique.

## Introduction and background

In this new era of the renaissance of non-penetrating glaucoma surgeries, newer implants, and shunt procedures, the role of trabeculectomy (TRAB) as the gold standard of glaucoma procedures is ambivalent. Even though many practitioners claim that TRAB will not survive in the near future, it still remains the first choice for most glaucoma surgeons. in cases with advanced damage, rapid progression despite maximal medical therapy, and in patients where the target intraocular pressure (IOP) required is very low. 'Trabeculectomy' procedure reported by Cairns in 1968 has undergone various modifications to increase outflow and achieve long-term success [1]. But the main issues with TRAB include short and long-term complications like hypotony, hypotonic maculopathy, wipe-out phenomenon, bleb leaks, cataracts, choroidal effusion, and hemorrhage [2]. These complications are accelerated with the concomitant use of anti-fibrotic, but without them, the chances of short-term failure are also relatively high [3,4]. The advent of novel minimally invasive glaucoma surgeries (MIGS) and non-penetrating surgeries (NPGS) have paved the path for lesser complicated yet effective ways of controlling IOP [5-8]. This review article summarizes the evolution and modifications of TRAB and its comparison of efficacy with neoteric shunt procedures while trying to answer whether TRAB has a future in the modern surgical world.

## Review

Evolution of filtering surgeries

**Figure 1:**
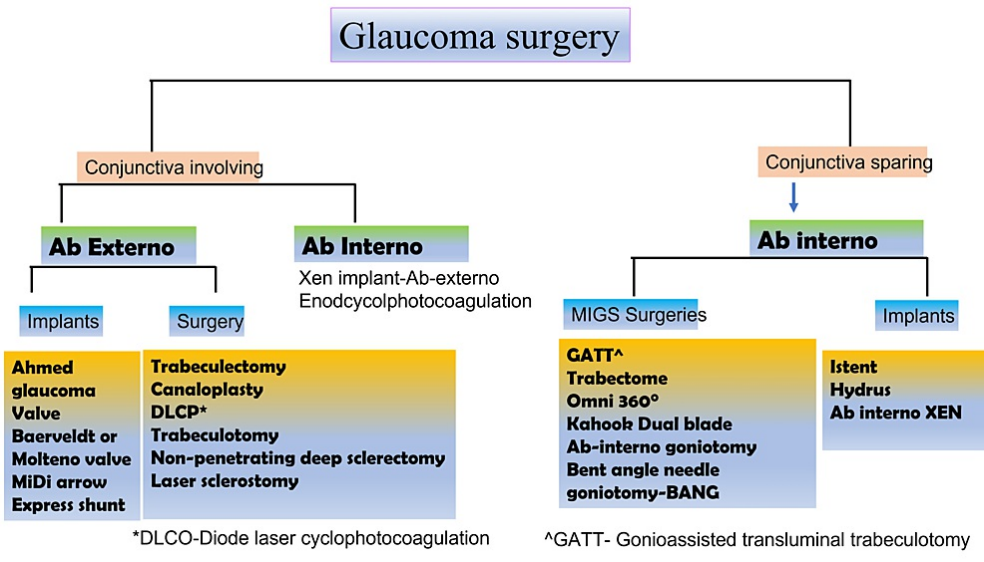
Schematic diagram illustrating different surgical options for the treatment of glaucoma

Glaucoma surgery now encompasses a variety of surgeries apart from conventional trabeculectomy (Figure 1).

Trabeculectomy underwent significant changes from an initial sclerotomy to an anterior sclerotomy, later a sclerectoiridectomy in 1906 or limbal trephination and iridencleisis to provide a permanent fistula by using iris as a wick between the anterior chamber and the subconjunctival space [2]. This was modified with a peripheral iridectomy and thermal sclerostomy (1958), or posterior lip sclerectomy before guarded filtration surgeries were introduced to offset the catastrophic complications with full-thickness procedures [2,8,9].

Evolution of partial-thickness procedures

Cairns JE initially described trabeculectomy in 1968 [1] that was later modified by Watson in 1970 [10,11]. Over the years, it has undergone modifications and supplementations to improve long-term success and reduce complications. Cairns described TRAB as a bypass procedure of making a deep scleral flap with excision of a small segment of the canal of Schlemm with trabecular tissue, Removal of the trabecular barrier at that point thus allowed an alternative resistance-free pathway. Few clinicians consider the name a misnomer, as cutting mainly the Schlemm's canal and adjoining corneal tissue will also serve the purpose, and clearing the trabecular tissue alone is not mandatory [11]. But the initial procedure was associated with complications of a full-thickness procedure and had high rates of failure [3,4,10-12].

Complications and the search for newer procedures

Early trabeculectomy filtering procedures were associated with a high rate of complications like hypotony, hypotonic maculopathy, choroidal detachment, suprachoroidal hemorrhage, bleb-related infections, and endophthalmitis [12-18]. Early cases of bleb leaks, shallow anterior chamber, and hypotony can be resolved with the use of large bandage contact lenses, pressure patching, symblepharon rings, and the Simmons shell. However, a flat anterior chamber with lens-corneal touch requires immediate surgical intervention to prevent rapid cataract development and irreversible corneal endothelial damage [12-14]. Initial studies have reported hypotony and choroidal detachment as late as 2-26 months following primary uncomplicated surgery that warrants a repeat surgery [14,15]. These complications forced surgeons to search for newer surgeries or ways to increase the safety profile while not compromising on the surgical success of trabeculectomy.

Partial-thickness procedures- new horizons

Watson and Barnett later modified this procedure by making a 5 x 5 mm partial-thickness flap and making a corneoscleral window for the passage of aqueous humor [10]. The original TRAB technique described by Cairns never intended to make a drainage bleb, but later it was observed that cases with good bleb had a higher success rate. It was then that the focus shifted to considering TRAB as a filtration surgery, and more attention was focused on the surgical techniques, which facilitated the creation of diffuse drainage blebs [16-18]. In the late 1960s, in order to create a track between the subconjunctival space and the anterior chamber, various methods of ab-interno and ab-externo approaches were tried using pulsed Nd: YAG laser, carbon dioxide laser, and excimer laser [16-19]. However, higher failure rates with laser surgeries make TRAB the standard procedure of choice for ensuring long-term success [19]. Newer procedures with comparable IOP outcomes are still evolving and are yet to replace TRAB as the gold standard for preservation of visual function in early-moderate glaucoma and more so for advanced stages of glaucoma, where TRAB still remains the surgery of choice.

Modifications in trabeculectomy

Antifibrotics to the Rescue

Increased use of anti-fibrotic agents like mitomycin C (MMC) and 5-fluorouracil (5-FU) along with TRAB started in the early 1990s to enhance the success rate, long-term survival rate, and decrease the progression of glaucoma [15,19-26]. In recent years, the use of MMC has significantly increased, while that of 5-FU has declined as a preferred practice pattern for primary TRAB [19-22]. A United Kingdom survey recently reported the use of anti-fibrotic agents in primary TRAB in 93% of their cases, of which 63% used 5-FU and 97% used MMC [20]. Various doses and duration of MMC use have been tried to offset delayed complications like bleb thinning, bleb leaks, or endophthalmitis. The American Glaucoma Society survey in 2016, claimed the dosage of MMC as 0.4 mg/mL (ranging from 0.2 to 0.5 mg/mL) applied for 2 minutes (range 45s-3 minutes) for primary TRAB, as the most popular and safer method [20]. Considering the role of angiogenesis in TRAB failure by wound modulation, the use of anti-VEGF agents is being tried in place of antifibrotics in TRAB. Liu et al. [21] reviewed eight randomized control trials (RCT) on TRAB with bevacizumab and concluded that bevacizumab and MMC had similar efficacy in IOP reduction. However, bevacizumab has been associated with a higher risk of leaking bleb and encystation, with other major issues being cost-effectiveness and off-label use [22]. The most recent RCT on intracameral bevacizumab in TRAB showed comparable surgical efficacy and IOP reduction to MMC, but with an increased rate of bleb leaks [23]. Recently the use of Ologen collagen matrix has been found to effectively modulate fibrous tissue formation thus decreasing the chances of failure [24,25]. Few surgeons have also tried using a combination of Ologen and MMC, with encouraging results [25]. A five-year follow-up study comparing Ologen to MMC also showed comparable results in both efficacy and safety between the two groups [26]. However, the cost of the Ologen implant is a major limiting factor for developing countries.

How Trabeculectomy Lost the Battle

Though TRAB success rates improved with the use of antifibrotics, the rates of delayed complication rates also increased parallelly, which again questioned the efficacy of TRAB as a standard glaucoma filtering surgery [15-18,27-30]. Belyea et al. studied 385 eyes that underwent TRAB with antifibrotics (MMC and 5-FU) and found an incidence of late repetitive multifocal bleb leaks in 1.8% of the eyes [15]. The incidence was equal among the two antifibrotics according to their study. The median period of the presentation was 20 months post-surgery. Singh et al. [27] studied the complications associated with the use of 0.2 mg/ml of MMC in TRAB and reported late bleb leaks, scleral necrosis, and hypotonic maculopathy as the major complications. It is now understood that their use results in the formation of thin and avascular blebs even in the delayed postoperative period, paving way for the easier migration of pathogens across the bleb and increased chance of delayed-onset endophthalmitis and blebitis. Incidence of bleb relation infection with MMC TRAB procedures reduced from 5.7% to 1.2% after the 1990s, after the introduction of MMC into clinical practice [28]. A recent study by Vaziri et al. [29] reported the incidence of endophthalmitis post trabeculectomy to be 0.45 ± 0.2% for confirmed cases and 1.3 ± 0.34% for confirmed plus presumed cases. The most common microbiological flora isolated from eyes with bleb-related infections includes *Staphylococcus aureus*, coagulase-negative staphylococci, Corynebacterium, and *Haemophilus influenza* [30].

Safe TRAB Re-emergence and Renewal

Since there was an increasing understanding of the causes of MMC-related bleb complications, safer techniques were now sought to prevent these delayed complications [15,18,30-31]. Khaw et al. [31]. designed a range of strategies commonly known as Moorfield's safe surgery techniques to improve the control of IOP as well as to preserve visual acuity by minimizing bleb-related complications and hypotony. Three major objectives in the adoption of the technique include: 1) prevention of hypotony; 2) prevention of thin uncomfortable cystic blebs and 3) prevention of limbal leaks of aqueous. Various steps adopted to prevent hypotony include a fornix-based conjunctival flap, making a small sclerotomy punch, continuous intraoperative anterior chamber infusion to achieve optimal pressure titration and to prevent hypotony, posterior placement of the MMC loaded sponges ensuring posterior flow, avoidance of >3minutes of MMC application at any single time, and a thorough wash of the area after each application. To prevent cystic uncomfortable blebs, selection of a superior location under the eyelid, larger area of treatment, fornix-based flap to minimize posterior scarring, and posterior diversion of aqueous by altering scleral flap construction, are some useful measures for safer TRAB with lower complication rates. Adopting a corneal groove-closure technique also helps in preventing limbal leaks of aqueous. Adoption of these techniques reduced the delayed complication rates associated with MMC use and this ushered in a resurgence of TRAB in glaucoma until the advent of technologically assisted filtering procedures [31].

Technological advancements supersede trabeculectomy

Minimally Invasive Glaucoma Surgery (MIGS)

**Figure 2:**
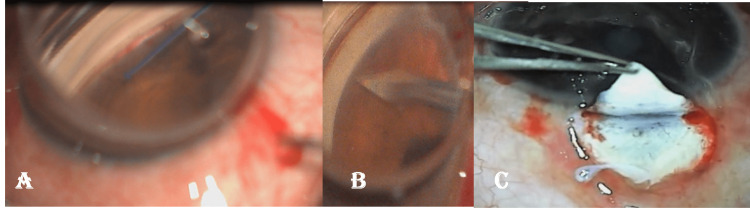
Comparison of open glaucoma surgery versus minimally invasive glaucoma surgeries A- showing intraoperative gonioscopic assisted transluminal trabeculectomy being done with 5-0 prolene suture through a needle track entry into the anterior chamber. B- shows ab-interno goniotomy. C- shows routine trabeculectomy being done that entails cutting open part of the trabecular tissue All images are from the corresponding author's own work.

Those procedures wherein the trabecular meshwork (TM) is incised /excised under direct supervision using specialized instruments are called ab-Interno or microinvasive glaucoma surgery [32-38]. These include the usage of trabectome, kahook dual blade, microhook ab-interno trabeculectomy, gonioscopy assisted transluminal trabeculotomy (GATT, Figure 2A), ab-interno goniotomy (Figure 2B), and ab-interno trabeculotomy 360 degrees. These are usually not associated with a bleb, require smaller incisions of entry, and are therefore not associated with bleb-related complications (Figures 1-2). A meta-analysis found the success rate of trabectome alone to be 46%, and when combined with phacoemulsification to increase to 85%, both achieving >30% IOP reduction [32]. With gonioscopy-assisted transluminal trabeculotomy (GATT), results have shown an IOP decrease of approximately 7.7 mmHg and 11.1 mmHg at 6 and 12 months, respectively. The number of anti-glaucoma medications (AGMs) reduced by 0.9 and 1.1 on average at 6 and 12 months [33]. Similarly, trabeculotomy 360 procedures performed on patients with refractory primary open-angle glaucoma (POAG) reported a 20% IOP reduction in 59% of patients, with the average number of anti-glaucoma medications dropping from 1.7 ± 1.3 to 1.1 ± 1.0 medications [34]. However, this had a 25% failure rate, with the majority requiring a second procedure within 12 months. Another study comparing ab-interno trabeculectomy with trabectome with ab-externo trabeculectomy found a lower success rate (22.4% Vs 76.1%), with 43.5 % requiring a second procedure for effective IOP control [35]. Even now, these procedures are used for moderate to early glaucoma, while TRAB remains the time-tested surgery for advanced glaucoma. Further, none of these procedures have been reported to offer long-term preservation of visual function better than TRAB or to be cost-effective for the patient in developing countries.

Non-penetrating glaucoma surgeries (NPGS)

Metanalysis comparing TRAB and non-penetrating glaucoma surgeries (NPGS) has concluded that TRAB results in much better control of IOP than NPGS [28-32]. Though the complications rates with TRAB are higher, it is preferable in cases with advanced chronic glaucoma and pseudoexfoliation glaucoma, where NPGS offers a lower success rate [32]. In 1964, Krasnov published his first report on a novel technique called "sinusotomy," which consisted of removing a lamellar band of the sclera and opening the Schlemm's canal over 120° [36]. He believed that the aqueous outflow resistance was mainly at the level of scleral aqueous drainage veins and not in the trabecular meshwork. Hence, no superficial scleral flap covered the sclerectomy in this technique. However, the sinusotomy procedure was eventually abandoned due to the difficult learning curve and less reduction in IOP compared with TRAB.

In 1989, Fyodorov and Kozlov described another technique called deep sclerectomy. In this procedure, careful scraping of the Schlemm's canal bed is done to remove a homogenous "external trabecular membrane" that allows aqueous humor to exit through the remaining inner trabecular layers [37]. Later in 1999, Stegmann et al. [38] reported 'viscocanalostomy' where a high molecular weight viscoelastic substance is injected into the opening of Schlemm's canal to enlarge the canal. This procedure allows the aqueous to bypass the juxtacanalicular trabecular meshwork and also drains the aqueous from the exposed Descemet's window. These surgeries were designed as a safer alternative to reduce complications of a full penetrating procedure while allowing filtration through the Schlemm's canal. 

Trabecular bypass and suprachoroidal procedures

Micro-invasive glaucoma implants, targeting the conventional outflow pathway, have emerged in the field of glaucoma over the last two decades to address an unmet need for better therapeutic options [32-45]. Various approaches have been adopted by these procedures to bring down the IOP by directly bypassing the trabecular meshwork, dilating the Schlemm's canal, and enhancing the uveoscleral outflow by assessing the suprachoroidal space and decreasing the aqueous production by ablating the ciliary body. One study reported a mean reduction of 7.0 ± 4.0 mmHg with I-stent combined with phacoemulsification versus a mean IOP reduction of 5.4 ± 3.7 mmHg with phacoemulsification alone, with 84% of the former eyes being medication free [39]. Another trial evaluated the safety and efficacy of CyPass® stunt (ab-interno-supraciliary space shunt) and reported a higher reduction of IOP (77% vs 66%) in eyes that underwent stent implantation along with cataract surgery. Furthermore, 85% of eyes in the CyPass® group were medication-free at two years [40]. However, the device was later withdrawn due to certain safety concerns over follow-up [41]. Gabbay and Ruben did a retrospective analysis on the safety of CyPass® stents and reported few other adverse effects over a short follow-up like postoperative pressure sikes (28%), hyphema, vitreous hemorrhage, choroidal effusion, and retinal detachment [42]. A hydrogel implant (XEN) is a newer FDA-approved implant that helps in shunting aqueous outflow into the subconjunctival space. Studies have reported a >20% reduction in IOP in 75.4% of patients, with a decrease in an average number of AGMs from 3.5 to 1.7 at 12 months postoperatively [43]. Studies comparing the latest XEN implant to conventional TRAB have claimed a higher and more efficacious IOP reduction with TRAB [44,45]. Though MIGS is now recognized as an alternative to TRAB, the major concerns include the steep learning curve and the varying safety profiles of different surgical procedures. Further, the cost-effectiveness, need for sophisticated machinery and instruments, and the need for frequent follow-up/additional surgeries, questions the actual effectiveness for visual function and the long-term applicability of these procedures worldwide.

Trabeculectomy in clinical practice

TRAB Versus Lensectomy 

Since cataract extraction alone was reported to cause IOP reduction, TRAB has been compared with cataract extraction alone in POAG and primary angle-closure glaucoma (PACG) [46-57]. Tham et al. [46] compared phacoemulsification (PHACO) alone to TRAB with MMC in medically uncontrolled angle-closure glaucoma without cataracts. Both groups resulted in significant and comparable IOP reduction at 24 months after surgery (IOP reduction of 34% for PHACO alone vs. 36% for TRAB+MMC, {P=0.76}). Nevertheless, TRAB+MMC-treated eyes required fewer AGMs than PHACO alone eyes. The same group studied the effect of combined phaco trabeculectomy (PT) to phacoemulsification alone (PHACO) and claimed that the former procedure is more effective than PHACO alone group in controlling IOP in medically uncontrolled chronic angle closure glaucoma eyes with coexisting cataract [47]. However, the PT group was associated with more surgical complications. An analogous study on POAG patients was done by Takihara et al. [49] and they concluded that TRAB with MMC in pseudophakic eyes post phacoemulsification is less successful compared with that in phakic eyes. However, no significant intergroup difference was noted in the number of postoperative antiglaucoma medications, surgical complications or in the number of laser suture lysis procedures.

TRAB Combined Cataract and Glaucoma Surgery

There had been numerous studies comparing TRAB with phaco trabeculectomy with anecdotal results (Table 1) [50-57]. However, a recent metanalysis on phaco trabeculectomy (PT) versus TRAB (TRAB) with or without later phacoemulsification did not find a significant difference in IOP reduction between the two procedures. A total of 25 studies were included comprising 2315 eyes that underwent PT and 2216 eyes that underwent TRAB, wherein, PT was associated with a lower risk of postoperative complications and better best-corrected visual acuity (BCVA) compared to TRAB [50]. Li et al. also evaluated the effect of PT versus TRAB alone and concluded that the PT group had better outcomes when compared to TRAB. However, the sample size and the follow-up period were less in their study [51]. Contrary to this Lochhead et al. stated that TRAB was better with a significant difference in the IOP reduction and surgical success when compared to PT [52]. Chang et al. [53] compared the effect of PT with 5-FU to TRAB with 5-FU and found conflicting results wherein the surgical success rate was similar for both, with a greater mean IOP reduction in the latter. Choy BN asserted an equal IOP control with the TRAB group having more diffuse blebs and less incidence of failure [54]. Another study by Tan et al. gave contrasting results with a higher rate of complications in the TRAB group than in the PT group [55]. Lam and Wechsler found comparable IOP reduction in both eyes at 5 years with both procedures, though, the number of AGMs required postoperatively was higher in the PT group [56].

**Table 1:** Studies comparing outcomes of phacotrabeculectomy with trabeculectomy alone. IOP- Intraocular pressure; POAG- primary open-angle glaucoma; PACG- primary angle closure glaucoma; PXG- pseudoexfoliative glaucoma; PDG- pigmentary glaucoma; NTG- normal-tension glaucoma, CACG- chronic angle closure glaucoma, SEC- secondary glaucoma; MMC-mitomycin C; 5-FU-5-fluorouracil; PT-phaco trabeculectomy; TRAB- trabeculectomy Li et al. [51]; Lochhead et al. [52]; Chang et al. [53]; Choy BN [54]; Tan et al. [55]; Lam and Wechsler [56]; Graf et al. [57]

Study author (Ref)	Method	Type of glaucoma	Parameters compared (n)	Outcomes measured	Limitation	Conclusion
Li et al.	Retrospective review	POAG	Phacotrabeculectomy (49) Trabeculectomy (65) MMC	(1) IOP (12 months) (2) Visual acuity (12 months) (3) Complete success (IOP ≤ 21) (4) Qualified success (IOP > 21 mmHg, but decreased to ≤21 mmHg after taking IOP-lowering medication)	-Small sample size -Short follow up	PT better than TRAB
Lochhead et al.	Retrospective	POAG	Phacotrabeculectomy (44) Trabeculectomy (44) No anti-metabolite used	(1) IOP (12 months) (2) Complications (3) Anti-glaucomatous medication (4) Surgical success	-Retrospective	Mean IOP reduction and surgical success significantly lower in PT group
Chang et al.	Retrospective study	POAG PACG CACG PXF PDG	Phacotrabeculectomy (45) Trabeculectomy (47) 5-FU	(1) IOP (minimum 12 months) (2) Surgical success (3) Anti-glaucomatous medication (minimum 12 months)	-IOP reduction was greater with TRAB, -Surgical success was similar, still authors recommend PT over TRAB	Similar success rate
Choy BN	Retrospective	POAG CACG Uveitic-glaucoma	Phacotrabeculectomy (20) Trabeculectomy (18) no anti-metabolite	1) IOP (3 months) (2) Complete success (IOP	-Short follow up period -Non-homogenous population and baseline characteristics	Equal IOP control with both procedures Diffuse bleb formation, lesser incidence of hypotony and failure with TRAB.
Tan et al.	Retrospective	POAG PACG	Phacotrabecuelctomy (334)-PACG;608-POAG) Trabeculectomy(112-PACG;208-POAG)	Complications (12 months) Needling	-Mixed races included -Non-uniform use of antimetabolites	PT less complication than TRAB, No difference between POAG and PACG
Lam and Wechsler	Retrospective	Not mentioned	Phacotrabeculectomy-(44) Trabeculectomy-(79)	1)IOP 2)number of glaucoma medications, treatment success rates	Type of glaucoma not mentioned Retrospective study—chances of selection bias Non -standardised procedures and use of MMC Multiple surgeons involved	IOP reduction similar fewer supplemental glaucoma medications in TRAB group
Graf et al.	Prospective study	POAG PXG NTG CAG SEC	Phacotrabeculectomy (161) Trabeculectomy (85)	(1) IOP (24 months) (2) Complete success (achieved target pressure according to visual field defects)	Unequal sample size	Comparable results between the two procedures

TRAB Versus Tube Surgery

Implants have revolutionized glaucoma surgery, especially in refractory cases [58-65]. A recent metanalysis comparing five systematic reviews on TRAB versus shunt surgeries concluded that shunt surgeries might achieve greater qualified success than TRAB [58]. It is, however, not clear whether the aqueous shunts are superior to TRAB owing to the lack of sufficient evidence with regards to aspects like cost-effectiveness and long-term visual function preservation. Studies comparing TRAB versus tube surgeries and their outcomes are listed in Table 2. Another meta-analysis by HaiBo et al. comparing Ahmed glaucoma valve implant (AGV) to TRAB also reported no significant difference in IOP reduction between the two surgeries [59]. Similarly, Tseng et al. [60] conducted a Cochrane database systematic review on the safety and efficacy of aqueous shunts (both Ahmed and Baerveldt implants) in comparison with conventional TRAB and concluded there were not many differences between aqueous shunts and TRAB for glaucoma treatment. A systematic review by Hong et al. [61] on glaucoma drainage devices (GDDs, including Ahmed, Molteno, Baerveldt, Krupin) with a total of 52 studies and 2682 patients, concluded that GDD is more effective in refractory glaucoma. To summarize, TRAB with MMC seems to be equally effective as tube-shunt surgeries with preservation of long-term visual function being achieved by both surgeries, albeit with TRAB achieving it for a longer time [63].

**Table 2:** Comparison of tube versus trabeculectomy IOP- intraocular pressure; TRAB- trabeculectomy; AGMs- antiglaucoma medications; AGV- Ahmed glaucoma valve; RCT- randomized control trials, GDD- glaucoma drainage devices Ordenes-Cavieres et al. [58]; HaiBo et al. [59]; Tseng et al. [60]; Hong et al. [61]; Minckler et al. [62]; Gedde et al. [63]

TRAB VERSUS SHUNT STUDIES Author (ref)	OUTCOME MEASURED	SAMPLE SIZE AND TYPE OF GLAUCOMA	CONCLUSION	LIMITATION
Ordenes-Cavieres et al.	IOP reduction Surgical success Safety profile Visual acuity Visual field deterioration	Compared 5 systematic reviews—total of nine studies; 4RCTs	Shunts might achieve greater qualified success than TRAB, but the certainty of the evidence was low to conclude its superiority in all other outcomes.	Heterogenous study population, one study based on pediatric glaucoma
HaiBo et al.	-%IOP reduction -Complete and qualified success) -Relative risks for safety profiles	6 RCTs comparing 249 AGV 258 TRAB	-No significant difference in IOP, AGM reduction, success rate, and safety concerns	-Type of glaucoma patient not clearly defined
Tseng et al.	-IOP reduction -LogMAR visual acuity -Adverse events -Quality of life	2099 patients	-Inconclusive evidence to report any difference between two procedures	Heterogenous study population -Heterogenous surgical procedures
Hong et al.	Surgical success	52 Studies including 2682 patients	-GDD are more effective in controlling refractory glaucoma	-Diverse etiology -Heterogenous surgical techniques and implants -Lack of uniformity in study designs
Minckler et al.	-IOP reduction -Complication rate Surgical success	17 RCTs that used Molteno 6 Baerveldt 3 AHMED 12 Krupin1	-Comparable benefits of shunts to TRAB in all complex glaucoma. -No advantages in adding antifibrotics to shunts -Clinical failure rate comparable to TRAB.	Heterogenous study group
Gedde eta al.	-IOP reduction -Success rate -Complication rate -Visual outcome	212 eyes comparing tube versus TRAB	-TRAB had greater IOP reduction than tubes. -Initial postoperative complications more in TRAB -Serious complications were similar in both groups.	-Multiple surgeons -No standardized definition of surgical complications

TRAB Versus Laser Trabeculoplasty

Laser trabeculoplasty has been a well-established technique for lowering IOP in POAG and ocular hypertension patients over the last two decades [66-70]. Wise and Witter reported IOP reduction by 10 mm Hg in 40 patients with phakic eyes, using argon laser, with 65% of these eyes requiring AGMs to control IOP [66]. The Glaucoma Laser Trial Research Group compared argon laser trabeculoplasty (ALT) to antiglaucoma medication and found better control in IOP with laser trabeculoplasty alone compared to a single AGM at 6 months, 1 year, and 2 years [67]. Studies evaluating selective laser trabeculoplasty (SLT) and glaucoma surgery are lacking in the literature. The EMGT (Early Manifest Glaucoma Trial) study observed that a 1 mmHg reduction in IOP from baseline decreased the risk of progression by 10% [68]. The advanced glaucoma intervention study (AGIS) looked at the effect of ALT before or after TRAB and found no change in white individuals. Neither prior ALT nor prior TRAB had a statistically significant effect on the failure of other procedures [69]. For 168 patients with uncontrolled chronic glaucoma, Migdal and Hitchings conducted a prospective clinical study comparing laser trabeculoplasty, medical therapy, and surgery as the primary therapy and concluded that the surgical group had the lowest average intraocular pressures and was the most successful at managing IOP diurnal swings [70]. Whilst laser trabeculoplasty resulted in a smaller reduction in pressure, these individuals were more likely to have high-pressure spikes.

TRAB in current glaucoma practice

TRAB In POAG

The role of TRAB in primary open-angle glaucoma patients (POAG) is well-established, however, there had been few anecdotal reports from few studies on its IOP reduction rates and visual field progression rates [71-75]. A recent study by Mataki et al. [71] in POAG documented a visual field (VF) progression of 0.7 decibels (dB)/year with a mean IOP of 15.7 mmHg. Similarly in a US-based study, Iverson et al. [72] reported a VF progression rate of 1.1 dB/year pre-operatively that had a mean IOP of 13.5 mmHg. Caprioli et al. [73] also confirmed in their study that TRAB can improve or maintain long-term visual function, a result that has not been proved unequivocally with other newer or older glaucoma surgeries.

TRAB In PACG

TRAB is the most common procedure used to reduce the IOP in both acute primary-angle closure glaucoma and chronic primary-angle closure glaucoma that are unresponsive to medical and laser treatment [76-77]. The overall success rate of TRAB varies from 68% to 100% depending upon the race and population [77]. However, because of the complications associated with TRAB, including cataract development, this is now less preferred. Adding to this is the high incidence of malignant glaucoma in this group of patients. 

TRAB in Pseudoexfolaition Glaucoma

Pseduoexfoliative glaucoma (XFG) is known to be more aggressive than other types of glaucoma with a high rate of intraoperative complications like vitreous loss, zonular damage, clinically significant choroidal detachment, and chor­oidal hemorrhage [78-81]. Popovic and Sjostrand [80] compared the efficacy of TRAB in XFG eyes and POAG eyes and reported comparable results in both with a marginally better outcome in XFG eyes. Contrary to this, a recent study by Li et al. proclaimed significantly lower long-term success rates at 3 years and 5 years of follow-up in XFG eyes than in POAG eyes, though the short-term success rates were similar [81]. Ehrnrooth et al. [78] compared 55 POAG eyes with 83 XFG eyes and found a significantly higher overall success rate for patients with POAG than XFG and reported that a higher preoperative IOP>30 mm Hg in the early postoperative period having an adverse effect on the surgical success of TRAB in XFG. Another study by Gurlu et al. [79] found no significant difference in the long-term success of TRAB between the two groups whose clinical characteristics are otherwise similar.

TRAB in Normal Tension Glaucoma (NTG)

Several landmark trials and studies have reported the efficacy of TRAB in NTG [82-87]. Naito et al. studied the effectiveness of TRAB in NTG patients with IOP<15 mmHg and found a significant reduction in mean IOP (8.1 ± 2.9 mmHg) and the number of AGMs (0.8 ± 1.5) [86]. In the Collaborative Normal-Tension Glaucoma Treatment Study (CNTGS) [83,84], nearly half of the eyes had undergone surgery, with an average post-operative IOP of 10.6 mmHg. The EMGT study [82], suggested that ALT may have a limited function in the treatment of NTG. A recent study that evaluated the effectiveness and long-term outcomes of TRAB using Moorfield’s technique claimed to have more successful long-term outcomes along with better safety and visual acuity preservation [87].

## Conclusions

TRAB for glaucomatologist-a friend or foe?

Thus, even amidst a myriad of newer surgical techniques, trabeculectomy still remains the preferred procedure, especially for those patients with advanced glaucoma and rapidly progressive disease. The ab-Interno procedures, MIGS, and newer microshunts are advised mainly in mild to moderate open-angle glaucoma cases and are relatively contraindicated in angle-closure cases. TRAB and implants/shunts still remain the rescue surgery in all refractory cases and inflammatory and neovascular glaucoma cases. MIGS and other ab-interno surgeries have expanded our options for treatment of mild to moderate stages of glaucoma, but fail to reach IOP levels at, or below the episcleral venous pressure achieved with a trabeculectomy. The IOP reduction achieved with trabeculectomy alone surpasses all the novel innovative methods. As our glaucoma population ages, there is an increasing need to titrate the IOP to a single-digit value that can be achieved only with TRAB compared to all novel operations. TRAB has been time-tested in terms of long-term success in a reduction in IOP, surgical success, and the preservation of visual fields. The evolution of safe surgical techniques and optimization of antifibrotic use has reduced the complication rate and increased the surgical success rates for TRAB. For those who have predicted the demise of trabeculectomy, we conclude that trabeculectomy does have a future and will remain the go-to procedure in most advanced and refractory cases in the coming decades.
